# Sonification of Deproteinized Bovine Bone Functionalized with Genistein Enhances Bone Repair in Peri-Implant Bone Defects in Ovariectomized Rats

**DOI:** 10.3390/jfb15110328

**Published:** 2024-11-05

**Authors:** Nathália Dantas Duarte, Gabriel Mulinari-Santos, Fábio Roberto de Souza Batista, Marcelly Braga Gomes, Naara Gabriela Monteiro, Ana Cláudia Ervolino da Silva, Reinhard Gruber, Paulo Noronha Lisboa-Filho, Pedro Henrique Silva Gomes-Ferreira, Roberta Okamoto

**Affiliations:** 1 Department of Diagnosis and Surgery, Araçatuba School of Dentistry, São Paulo State University “Júlio de Mesquita Filho”, Araçatuba 16015-050, São Paulo, Brazil; nd.duarte@unesp.br (N.D.D.); fabio.rs.batista@unesp.br (F.R.d.S.B.); naara.monteiro@unesp.br (N.G.M.); ana.ervolino@unesp.br (A.C.E.d.S.); 2 Department of Basic Sciences, Araçatuba School of Dentistry, São Paulo State University “Júlio de Mesquita Filho”, Araçatuba 16018-805, São Paulo, Brazil; marcelly.braga@unesp.br; 3 Department of Oral Biology, University Clinic of Dentistry, Medical University of Vienna, 1090 Wien, Austria; reinhard.gruber@meduniwien.ac.at; 4 Department of Physics, Bauru School of Sciences, São Paulo State University “Júlio de Mesquita Filho”, Bauru 17033-360, São Paulo, Brazil; paulo.lisboa@unesp.br; 5 Department of Dentistry, Ourinhos University Center, Ourinhos 19909-100, São Paulo, Brazil; pedro.gomes@unifio.edu.br

**Keywords:** biocompatibility, bone regeneration, dental implants, genistein, osseointegration, osteoporosis, rat, tibia

## Abstract

Estrogen deficiency is one of several contributing factors to catabolic changes in bone surrounding dental implants, impairing bone repair in defects requiring bone regeneration. Functionalizing bone substitutes is an alternative approach among various strategies to address this challenge. In this study, the aim was to evaluate the effect of functionalizing deproteinized bovine bone (Bio-Oss^®^, BO) with genistein via sonication on peri-implant bone defects in ovariectomized rats. The animals were randomly distributed according to the treatment into the following four groups (n = 10): BO sonicated with genistein (BOS + GEN), BO sonicated alone (BOS), untreated BO (BO), and blood clot only (CLOT). After twenty-eight days, implant removal torque was determined, and the peri-implant bone parameters were calculated based on computed microtomography. Additionally, the gene expression of bone turnover markers was evaluated. As a main result, the functionalization with genistein increased implant removal torque and the peri-implant bone volume in the BOS + GEN group compared to both BOS and BO groups (both *p* < 0.05). These findings suggest that the sonification of deproteinized bovine bone functionalized with genistein improves bone repair in peri-implant bone defects in ovariectomized rats.

## 1. Introduction

Estrogen deficiency is one of the various contributing factors to catabolic bone metabolism, leading to increased bone resorption in postmenopausal women [1,2]. Osteopenia is characterized by reduced bone density, particularly affecting the trabecular bone, which becomes thinner and loses its structural integrity [3]. This condition can progress to osteoporosis with its clinical hallmarks being fragility fractures [4]. Estrogen plays a crucial role in controlling bone remodeling and consequently maintaining bone mass [5]. Osteoporosis is not a contraindication to dental implant treatment; however, it increases marginal peri-implant bone loss [6]. Therefore, since estrogen deficiency is a contributing factor capable of causing catabolic changes in the peri-implant bone [2,3,4,5], it is necessary to ensure the peri-implant bone quality and consequent dental implant survival.

Dental implants are frequently placed in areas of alveolar ridge defects, dehiscence, or fenestrations, and bone grafts may be required to fill these peri-implant bone defects [7]. Likewise, bone substitutes are crucial for alveolar ridge preservation, proper implant stability, and bone formation for osseointegration [8]. However, the challenges of bone repair increase with estrogen deficiency [7]. This condition can reduce the differentiation of mesenchymal cells, impair angiogenesis [9], and increase osteoclast activity [10]. Estrogen deficiency may compromise an adequate peri-implant bone repair [11,12]. Also, estrogen deficiency can impair bone regeneration by lowering differentiation and maturation of osteoblasts and the formation of bone matrix [13,14]. Hence, it might be helpful to support the consolidation of bone substitutes in bone defects around dental implants under osteoporotic conditions.

Bio-Oss^®^ is an established bone substitute prepared from deproteinized bovine cancellous bone with the expected osteoconductive properties [15,16,17]. Despite Bio-Oss^®^ being widely used clinically, stimulating the local bone regeneration is advantageous for its consolidation with the implant and the local bone, particularly in situations of estrogen deficiency [18]. A promising alternative approach among various strategies involves the functionalization of Bio-Oss^®^ with biomolecules to enhance bone regeneration [19]. Biomolecules to enhance bone regeneration using bone substitutes are being verified in estrogen deficiency conditions [20]. Ideally, these biomolecules should overcome the catabolic circumstances of estrogen deficiency.

Genistein (C_15_H_10_O_5_) is a 7-hydroxy isoflavone categorized as an abundant isoflavone from soy [21,22]. This phytoestrogen has a molecular similarity to estrogen [23] and enhances osteoblast differentiation through estrogen receptors [24,25]. Its protective effect on bone is indicated by increasing the expression of osteocalcin [26], in addition to inhibiting osteoclastogenesis by changing the ratio of osteoprotegerin and receptor activator of nuclear factor kappa-B ligand [26,27]. Even though the ability of genistein to support bone regeneration under osteoporotic conditions was not confirmed [27], genistein is an effective treatment for overcoming experimental osteoporosis [25,28]. Our strategy is to distribute genistein within Bio-Oss^®^ equal particles in functionalization by sonification.

Sonification is a novel tool for developing nanoscale biomaterials [7,29]. It uses ultrasound to homogenize and distribute biomolecules, thereby amplifying their physical–chemical properties [30]. Consequently, nanoparticles can improve the cellular interaction in bone, enhancing osteoblast differentiation [29,31]. Sonification is also effective for incorporating biomolecules into bone substitutes [32]. Therefore, in this study, it was investigated how genistein incorporated into Bio-Oss^®^ by sonification affects bone repair in peri-implant defects using ovariectomized rats as a model for estrogen deficiency.

## 2. Materials and Methods

### 2.1. Ethics and Sample Size Calculation

The Ethics Committee approved this research on the use of animals from Araçatuba School of Dentistry, São Paulo State University (FOA/UNESP) under number 0499-2022. Additionally, this research was performed according to the ARRIVE Guidelines [33]. All evaluations were performed under calibration and blinding examination. The sample size of this study was calculated using the power test (OpenEpi, Version 3, Open-Source Calculator), based on results already published with a similar methodology using the values of the removal torque [7]. The means used for the calculation were 4.4 and 6.0 Ncm, with standard deviations of 1.51 and 2.6, with a significance level of 5% and power of 95% in a one-tailed hypothesis test. Therefore, the number of animals enrolled per group in this study was 10. Accordingly, a total of forty female six-month-old animals (*Rattus norvegicus albinus*, Wistar) weighing approximately 300 g were maintained at the Central Bioterium of Araçatuba School of Dentistry from UNESP in temperature-controlled conditions and exposed to a 12 h light–dark cycle. All the animals were kept in cages with water ad libitum and regular feed (Nuvilab, Curitiba, Brazil).

### 2.2. Estrous Cycle

The determination of the estrous cycle was executed before ovariectomy. The ovariectomy was performed on day 0. The peri-implant bone defects and implant placement were performed 30 days after ovariectomy, and the animals were euthanized 28 days after implant surgery. The animals were placed in individual cages for the estrous cycle evaluation, which was conducted daily [34]. To this, one or two drops of saline were introduced into the vagina, which was then aspirated and placed on a histological slide for immediate microscopic analysis to recognize the four phases of the estrous cycle, which are described as proestrus, estrus, metestrus, and diestrus [35]. After three regular cycles, the animals were applied for ovariectomy surgery.

### 2.3. Ovariectomy

Animals were kept on a six-hour preoperative fast. Then, the animals were subjected to sedation and anesthesia through intramuscular administration of ketamine hydrochloride (70 mg/kg; Francotar^®^, Virbac, São Paulo, Brazil) combined with xylazine hydrochloride (6 mg/kg; Xilazin^®^, Syntec, Barueri, Brazil). Trichotomy was performed in the abdominal region, followed by antisepsis with a povidone-iodine solution (10%; Rioquímica, São José do Rio Preto, Brazil). An incision was made with a No. 15 blade (Feather Industries, Tokyo, Japan) mounted on a n^o^ 3 scalpel handle (Hu-Friedy, Frankfurt, Germany) in the middle part of the abdomen. After accessing the peritoneal cavity, the adipose tissue was retracted to identify the uterine tube and ovary, which were then collected. The surgical wound was sutured with polyglactin 910 4.0 thread (Ethicon Inc., São José dos Campos, Brazil). Intramuscular antibiotic therapy post-surgery was administered to all animals with benzathine penicillin G (0.2 mL; Pentabiótico^®^ Veterinário Pequeno Porte, Fort Dodge Saúde Animal Ltda., Campinas, Brazil). Analgesia was provided intramuscularly for three days with sodium dipyrone (50–600 IU/kg; D-500^®^, Zoetis, Parsippany-Troy Hills, NJ, USA) and Tramadol (5 IU/kg; Nulli^®^, Ourofino Saúde Animal, Cravinhos, Brazil). One week before implant surgery, the estrous cycle was checked to evaluate the success of the ovariectomy, which remained in the diestrus phase. This same surgical technique followed a previous study [36].

### 2.4. Experimental Groups

After ovariectomy, the animals were randomized through a computer-generated list created in Stata 9.0 (StataCorp, College Station, TX, USA) and divided accordingly into the following four experimental groups (n = 10): CLOT control group, peri-implant defect filled with spontaneous blood clot formation; BO group, peri-implant defect filled with BO (Bio-Oss^®^ Small 0.25–1 mm; Geistlich Pharma AG, Wolhusen, Switzerland); BOS group, peri-implant defect filled with BO sonicated alone; BOS + GEN group, peri-implant defect filled with BO and genistein (Sigma-Aldrich, San Luis, CA, USA) sonicated.

### 2.5. Sonification and Functionalization with Genistein

The sonification was performed at the Advanced Materials and Nanotechnology Laboratory, School of Sciences from UNESP. First, the calculation to determine the volume of biomaterial in the bone defect was performed by the physics P.N.L-H. The peri-implant defect created measured 3 mm in diameter and 3.5 mm in depth with a volume of 12.56 mm^3^. It was subtracted by the implant volume of 5.25 mm^3^, measuring 1.5 mm in diameter × 3.5 mm in height. The total volume of the peri-implant bone defect measured 7.31 mm^3^. The underlying formula was volume = π.r^2^.a. Thus, the result of the total volume of BO for the peri-implant bone defect was 25 mg. BO untreated was used in the BO group, while BO sonicated alone was utilized in the BOS group, and BO sonicated together with 3 mg of synthetic genistein (≥98% purity; Sigma-Aldrich, San Luis, CA, USA) was used in the BOS + GEN group. The samples of BO were weighed as 25 mg on a precision scale (LW303iH, BEL Engineering^®^, Monza, Italy). Sonification was performed with a Sonics^®^ VCX-750 (Sonics & Materials, Newtown, CT, USA) at 750 W and 20 kHz for 15 min. A variable amplitude of up to 40% with ultrapure water Milli-Q^®^ (Millipore, Burlington, MA, USA) was used for the cavitation bubbles with high solute vapor. The time protocol was three cycles of three minutes each. Later, the bone substitutes were dried at 70 °C (TE-394/2 Model, Tecnal Scientific Equipment, Piracicaba, Brazil). The sonification followed a method as previously described [37]. Samples were sterilized using ultraviolet light for 20 min before the implant surgery.

### 2.6. Peri-Implant Bone Defects and Implant Placement

First, a peri-implant bone defect was created using larger drills in the upper cortical and medullary bones. Next, the bone defect was filled according to each experimental group. Lastly, the implant was placed into the bone defect with anchorage in the lower cortical bone, following a previously successful rat tibia model [7].

For that, animals were fasted for six hours before the surgery and anesthetized by a combination of ketamine hydrochloride (70 mg/kg; Francotar^®^, Virbac, São Paulo, Brazil) and xylazine hydrochloride (6 mg/kg; Xilazin^®^, Syntec, Barueri, Brazil). After anesthesia, a trichotomy was performed on the medial area of the right tibia. The antisepsis of the region was performed with a povidone-iodine solution (10%; Rioquímica, São José do Rio Preto, Brazil). A 1.5 cm incision was executed, and the tibial metaphysis was exposed through divulsion. In both tibias, a 1.3 mm bicortical drill was followed by a 3.0 mm drill in the upper cortical and medullary bone, thus maintaining the lower cortical to anchor the implant. Drilling was controlled by an electric motor (BLM 600 Model, Driller, Carapicuíba, Brazil) at a speed of 1000 rpm and contra-angle with 20:1 reduction (Angle Piece 3624N 1:4, Head 67RIC 1:4, KaVo, Biberach, Germany) under irrigation with sodium chloride solution (0.9%; Biosynthetic Ltda., Ribeirão Preto, Brazil).

The defects were filled with the respective biomaterials estimated in each group, except in the CLOT group which was left untreated. Finally, the implants were inserted in the middle of the bone defect. The implants were grade-four titanium (diameter of 1.5 mm and height of 3.5 mm; Titaniumfix^®^, São José dos Campos, Brazil) with a surface treated by a double acid attack using nitric, hydrofluoric, and sulfuric acid. Wound closure was performed with absorbable thread (Vycril 4.0, Ethicon; Johnson Products, São José dos Campos, Brazil) and monofilament thread (Nylon 5.0, Ethicon; Johnson, São José dos Campos, Brazil). In the immediate postoperative period, each animal received an intramuscular dose of 0.2 mL of penicillin G-benzathine (Fort Dodge Health Animal Ltda., Campinas, Brazil).

### 2.7. Euthanasia

After 28 days following implant surgery, euthanasia was performed according to a previous study [38]. Euthanasia was performed with an intraperitoneal overdose of sodium thiopental (2.5%; 150 mg/kg; Fort Dodge Saúde Animal Ltda., Campinas, Brazil) and lidocaine (2%; 10 mg/kg; Anesthetic; Laboratório Bravet Ltda., Rio de Janeiro, Brazil). The tibias of five animals per group were dissected for removal torque analysis. Next, the peri-implant tissue was collected for reverse transcription polymerase chain reaction (RT-PCR). The tibias of the other five animals per group were collected and stocked in 70% alcohol for micro-computed tomography (micro-CT). All implants were clinically without any signal of inflammation or infection, thereby no group presented any exclusion. 

### 2.8. Biomechanical Test (Removal Torque)

The removal torque was executed in five animals per group; thus, a counterclockwise movement was used to analyze the removal torque of implants from the bone using a digital torque wrench (TQ-680 Model; Instrutherm, São Paulo, Brazil) coupled with a critical implant (Conexão Sistemas de Próteses, São Paulo, Brazil). The maximum value of removal torque was recorded in Newton per centimeter (Ncm) until the implant rotated inside the bone, as in the previous study [36].

### 2.9. Micro-CT

The other five animals per group were used for micro-CT. The tibias were scanned using a SkyScan Microtomography (SkyScan 1272; Bruker, Aatselaar, Belgium) from the Multi-User Laboratory of Araçatuba School of Dentistry from UNESP. The images were captured on a camera with a pixel size of 12.45 mm, 2672 × 4000, using 8 μm-thick cuts with a 90 kV X-ray beam, a 111 μA current, a 0.5 mm Al filter, and a 0.4° rotation step. Afterward, the images were reconstructed using NRecon Software (SkyScan v1.6.9.8) with smoothing of 1, ring trace correction of 3, beam strength correction of 5%, and image correction variation of 0. 0 to 0.11. The images were appropriate in a new set of data, positioning all samples standardized in the transverse, longitudinal, and sagittal planes using Data Viewer Software (SkyScan 1.4.4; Leuven, Belgium). The analyses were established using CTAnalyzer Software (SkyScan CTAn, v1.12.4.0; Leuven, Belgium). A region of interest (ROI) between the third and fifth medullary implant threats was used to evaluate 100 slices from its most central portion. Images were converted to gray scale values between 25 and 90 shades representing the new bone formed, excluding the titanium implant and the biomaterials. The parameters evaluated were the percentage of bone volume per tissue volume (BV/TV; %) and the surface of intersection (IS; mm^3^), following the guideline [39]. The micro-CT analysis was used to characterize the tridimensional bone around the implants using CTvox Software (SkyScan 2.7; Leuven, Belgium). The micro-CT analysis was performed as recently reported [7].

### 2.10. Molecular Analysis (RT-PCR)

The same five tibias of removal-torque were immediately collected, ensuring at least 0.5 cm on each peri-implant side to preserve the bone in contact with the implant. RT-PCR evaluated the gene expression of proteins associated with bone repair. Each sample was washed in phosphate buffer solution and frozen in liquid nitrogen for total RNA extraction using Trizol reagent (Life Technologies Invitrogen, Carlsbad, CA, USA) and stored in a freezer at −80 °C. Following the RNA integrity, purity, and concentration assessment, cDNA was synthesized using 1 µg of RNA through reverse transcriptase reaction (M-MLV RT; Promega Corporation, Madison, AL, USA). The cDNAs from the samples were pipetted for the detection of genes with TaqMan Fast Advanced Mastermix (Applied Biosystems, Waltham, MA, USA) into a PCR plate (96 Well Fast Thermal Cycling; Thermo Fisher Scientific, Waltham, MA, USA). The RT-PCR was performed to assess the expression of osteoprotegerin (OPG; Tnfrsf11b; Rn00563499_m1), receptor activator of nuclear factor kappa-B ligand (RANKL; Tnfrsf1; Rn00589289_m1), osteocalcin (OCN; BGGLAP; Rn00566386_g1), and bone sialoprotein (IBSP; IBSP; Rn00561414_m1). The genes and their primer sequences are shown in Table 1. RT-PCR was performed using a Step One Plus detection system from Taqman (Applied Biosystems, Waltham, MA, USA) under the following conditions: 50 °C (2 min), 95 °C (10 min), and 40 cycles of 95 °C (15 s), 60 °C (1 min), followed by the standard denaturation curve. The expression was calculated relative to the expression of mitochondrial ribosomal protein following the ΔΔCT method with ß-actin (Actb Rn00667869_m1) as an endogenous control. The assay was performed in quadruplicate [7].

### 2.11. Statistical Analysis

Statistical analysis was performed using SigmaPlot 12.5 Software (Systat Software Inc., San Jose, CA, USA). After obtaining all the data, the homoscedasticity test (Shapiro–Wilk) was performed first. A significance level of *p* < 0.05 was considered to confirm the normality distribution of the data. Thus, a one-way ANOVA test was applied for the removal torque, microtomographic, and molecular data, followed by the Tukey post-hoc test for multiple comparisons. Also, a confidence level of *p* < 0.05 was considered significant.

## 3. Results

### 3.1. Removal Torque Values

**Figure 1:**
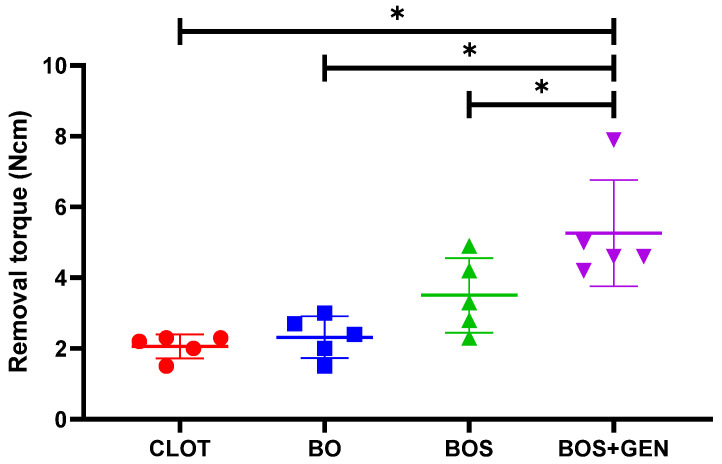
Biomechanical test of removal torque. Twenty-eight days after implant placement, each experimental group’s removal torque was tested. The removal torque was increased until the implant rotated into the bone, and the highest torque in Newton centimeters was recorded. The statistically significant difference in comparison to the BOS + GEN group (*p* < 0.05) is indicated by *, determined using one-way ANOVA followed by Tukey’s *t*-test.

The implants were successfully installed and stability verified through a removal test. Figure 1 shows that the BOS + GEN had the highest removal torque with 5.4 Ncm, compared to the BOS (*p* = 0.006), BO (*p* = 0.001), and CLOT (*p* = 0.0003) with 3.0 Ncm, 2.3 Ncm, and 2.1 Ncm, respectively.

### 3.2. Micro-CT

**Figure 2:**
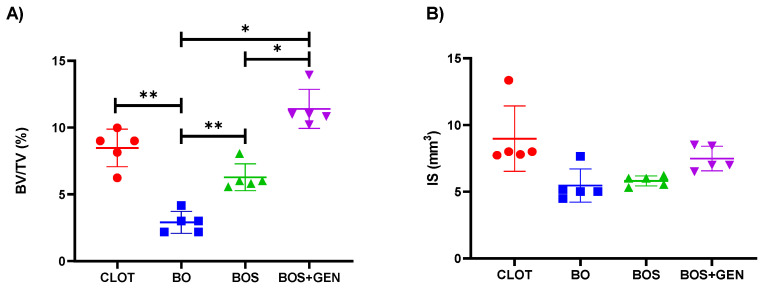
Micro-computerized tomography of peri-implant bone. Morphological parameters were estimated as follows: (**A**) percentage of bone volume per tissue volume (BV/TV, %); (**B**) intersection surface (IS; mm^3^). The statistically significant difference is indicated by * in comparison to the BOS + GEN group. ** denotes a statistically significant difference in comparison to the BO group. The *p* < 0.05 was determined using one-way ANOVA followed by Tukey’s *t*-test.

**Figure 3:**
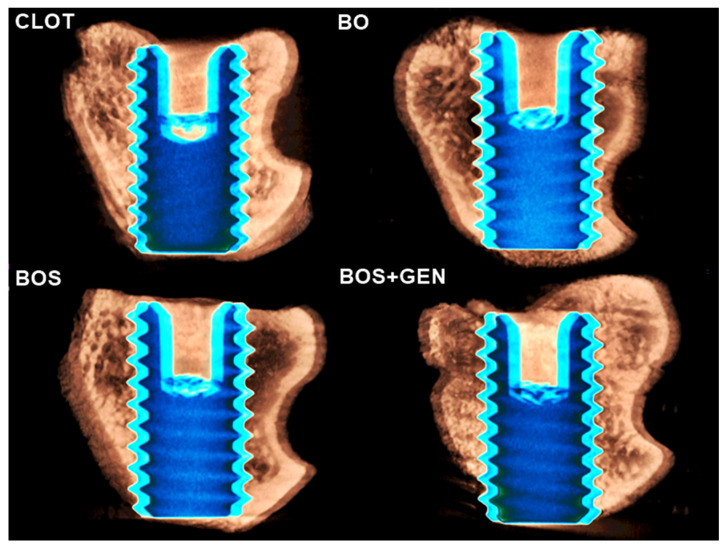
The 3D µCT reconstruction of the peri-implant bone area. The µCT images are representative of all four groups, CLOT, BO, BOS, and BOS + GEN, respectively. The microtomography images evidenced that genistein enhanced the peri-implant bone into the medullary implant threats in the BOS + GEN group. The microtomography was executed by applying the CTvox software (SkyScan, Version 2.7).

Consistent with the biomechanical outcomes, BV/TV was highest in the BOS + GEN group with 11.67% compared to CLOT (*p* = 0.104), BOS (*p* = 0.017), and BO (*p* = 0.0002) with 8.12%, 6.47%, and 2.32%, respectively Figure 2A. The IS parameter denoted bone-to-implant contact, and there was no statistical difference between the groups (*p* > 0.05); Tukey; Figure 2B. The representative three-dimensional image of bone repair around the implants installed can be observed in Figure 3.

### 3.3. Molecular Analysis

**Figure 4:**
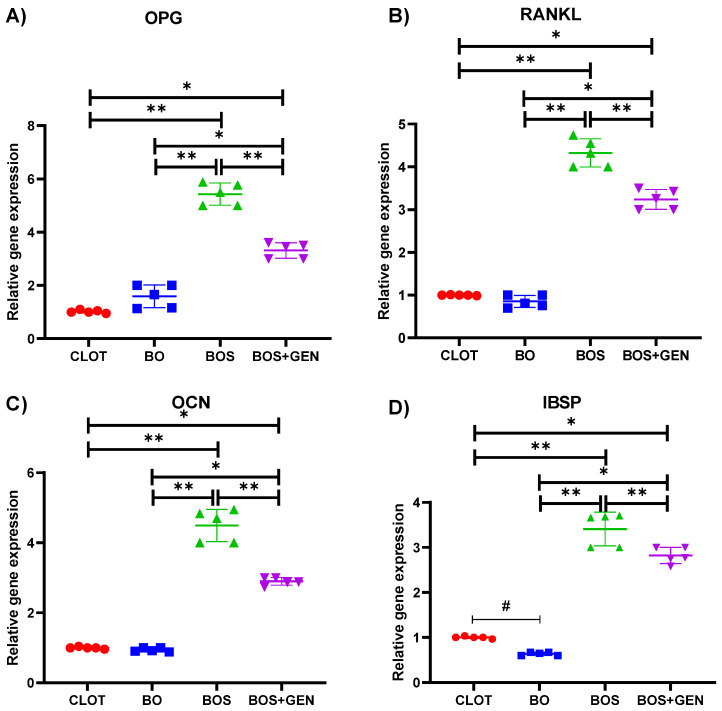
Molecular data from RT-PCR analysis of all groups. (**A**) OPG: osteoprotegerin; (**B**) RANKL: receptor activator of nuclear factor kappa-B ligand; (**C**) OCN: osteocalcin; (**D**) IBSP: bone sialoprotein. The * denotes a statistically significant difference in comparison to the BOS*GEN group. The ** denotes a statistically significant difference in comparison to the BOS group. The # denotes a statistically significant difference in comparison to the BO group. The statistically significant difference (*p* < 0.05) was determined using one-way ANOVA followed by Tukey’s *t*-test.

The relative gene expression of OPG, RANKL, OCN, and IBSP showed higher expression in the BOS and BOS + GEN groups, as shown in Figure 4A–D. Next are the CLOT and BO groups. Then, the statistical differences between the groups were BOS vs. CLOT (*p* < 0.0001; Tukey), BOS vs. BO (*p* < 0.0001; Tukey), BOS vs. BOS + GEN (*p* < 0.0001; Tukey), BOS + GEN vs. CLOT (*p* < 0.0001; Tukey), and BOS vs. BOS + GEN (*p* < 0.0001; Tukey). Besides the exact differences, the relative gene expression of IBSP included the statistical difference between CLOT vs. BO (*p* < 0.004; Tukey).

## 4. Discussion

Given the increasing life expectancy, dental implants for oral rehabilitation are increasingly necessary [40]. There is a growing population of postmenopausal women necessitating dental implants [40]. Low estrogen levels can compromise bone repair around implants [11,12], in addition to impairing bone regeneration [13]. There is consequently an interest in local therapies to enhance bone repair in peri-implant defects under estrogen deficiency [41]. To this end, we show here that the functionalization of BO by sonification with genistein, compared to all other experimental groups, increased the removal torque of implants in rat tibia. Consistently, µCT revealed a higher BV/TV with BOS + GEN compared to BO or BOS. The improved bone microarchitecture can justify the higher removal torque. In addition, RT-PCR analysis indicates increased bone turnover when sonification with genistein or sonification alone was tested. The RANKL–OPG ratio indicates bone activity [42], thus the constant ratio suggests that genistein has a low impact on osteoclastogenesis. On the other hand, higher levels of OCN and IBSP indicate enhanced osteoblast activity [43,44]. These findings suggest that incorporating genistein improves the consolidation of bone substitutes, leading to enhanced implant stability.

Previous studies have confirmed the positive effects of genistein on osteoblastogenesis [45,46]. Genistein-coated implants resulted in greater bone-to-implant contact in osteoporotic rats [45]. In this study, genistein was identified as a catalyst for enhancing osteogenesis and bone quality with a cohesive network of osteocytes around the implants [45]. Similarly, an in vitro study demonstrated that genistein stimulates osteoblast differentiation by upregulating the expression estrogen receptor-α gene and Runt-related transcription factor 2 [46]. In line with our findings, sonification of other bone substitutes also favored osteogenic differentiation [7,32]. Bioactive glass sonicated with teriparatide enhanced peri-implant bone repair in osteoporotic male rats [7]. Under physiological conditions, bioactive glass sonicated alone also increased peri-implant bone formation by upregulating WNT signaling [32]. Prior studies reinforced an increased cellular reactivity of sonicated nanomaterials mainly by their amplified physical and chemical effects [47,48]. Moreover, nanomaterials can raise the expression of bone proteins as enzyme alkaline phosphatase, Runt-related transcription factor 2 [49]. It can be speculated that genistein loaded by sonification can be an anabolic strategy for bone regeneration, effectively stimulating osteogenesis.

The present results are crucial to fully understanding the experiment. The CLOT group exhibited enhanced bone volume attributed to endogenous bone formed by the blood clot. The higher BV/TV in the CLOT group compared to the BO group can be explained by the void created by the absence of biomaterial within the defect. Furthermore, the quality of bone formation in the absence of bone substitutes remains uncertain, particularly with compromised estrogen levels [50]. Thus, although the CLOT group presented greater BV/TV than the BO and BOS groups, it showed the lowest torque value. Likewise, despite the higher torques observed in the BOS + GEN group, the IS parameter did not indicate a variance in bone-to-implant ratio in the groups. Notably, the biomaterial serves as a scaffold for osteoprogenitor cells, augmenting implant stability [4]. This outcome validates the biomechanical reinforcement of the peri-implant bone by the genistein and solicitation, as evidenced by the higher torque in the BOS + GEN and BOS group. However, there is no difference between the CLOT and the BO group when comparing the torque. Consequently, it is suggested that genistein or sonification alone increases the biomechanical results by augmenting the osteoconductive properties of the bone substitute.

The findings here should be considered within the limitations of this study. The major limitation is that we cannot conclude about the effects of genistein on bone quality. The absence of undecalcified histology is not stipulated in the present study, which could provide valuable perceptions of the bone cells present in each group. A further limitation of this study was that genistein was tested only in deproteinized bovine cancellous bone. The unique time of sonification is also a restriction; however, the sonification time of 15 min demonstrated the best results in our prior study [32]. Additionally, the long bones of rats do not apply for the alveolar bone repair. Moreover, examining at just a one-time point does not provide comprehension of the effects of genistein on bone remodeling. Finally, genistein is considered dose-dependent, where excessive systemic doses have been demonstrated to hinder bone formation [51]. The unique concentration tested provided beneficial results in our study. Thus, future investigations should consider using other bone substitutes and different sonification protocols and drug concentrations before moving to clinical studies.

This study has significant clinical relevance. The main clinical implication is that genistein can be applicable in different scenarios of bone regeneration to overcome estrogen deficiency. Genistein incorporated into deproteinized bovine bone offers a local therapy different from the systemic use of estrogen in postmenopausal women. Estrogen therapy is known to have potential complications in the treatment of postmenopausal women, including cancer and cardiovascular events [52]. Additionally, genistein avoids the side effects of antiresorptive drugs for osteoporosis, such as medication-related osteonecrosis of the jaw [53,54]. Importantly, the use of genistein did not demonstrate systemic or tissue toxicity as in previous in vivo studies [55,56]. Conversely, genistein positively stimulated bone cell proliferation [55]. Genistein also had an antibacterial activity, affecting its acid synthesis and cytoplasmic membrane [55]. From a clinical perspective, genistein could also be beneficial against the presence of microorganisms in bone defects. Thus, it could be interesting in the context of peri-implant diseases. However, the results of this research should be viewed within the circumstances of a pre-clinical animal study. Future studies with an in vitro focus should evaluate the effect of genistein on the biological behavior of osteoblastic cells underlying the formation of the mineralized bone.

## 5. Conclusions

In conclusion, the evidence presented here implies that sonification of deproteinized bovine bone functionalized with genistein improves bone repair in peri-implant bone defects in ovariectomized rats, enhancing implant stability. Thus, these preclinical data demonstrate that genistein, an anti-catabolic drug, can help enhance bone regeneration under osteoporotic conditions in a rat model.

## Figures and Tables

**Table 1:** Primer sequences used for RT-PCR analysis.

Gene	Gene Reference	Forward Primer, 5′ → 3′	Reverse Primer, 5′ → 3′
*OPG*	NM_057149.2	GCACTCCTGGTGTTCTTGGA	TTTGGTCCCAGGCAAACTGT
*RANKL*	NM_057149.1	CGAGCGCAGATGGATCCTAA	GAGCCACGAACCTTCCATCA
*IBSP*	NM_012587.2	GTACCGGCCACGCTACTTTC	ATCTCCAGCCTTCTTGGGTAGC
*OCN*	NM_013414.1	CTCTGAGTCTGACAAAGCCTTCAT	GTAGCGCCGGAGTCTATTCA
*ß-actin*	NM_031144.3	CCACCATGTACCCAGGCATT	CCTAGAAGCATTTGCGGTGC

## Data Availability

The authors will make the raw data supporting this article’s conclusions available upon request.
